# Effects of graded concentrations of supplemental lead on lead concentrations in tissues of pigs and prediction equations for estimating dietary lead intake

**DOI:** 10.7717/peerj.3936

**Published:** 2017-11-01

**Authors:** Kondreddy Eswar Reddy, Kyu Ree Park, Sung Dae Lee, Ji-Hyock Yoo, Ah Reum Son, Hyun-Jung Lee

**Affiliations:** 1 Animal Nutritional Physiology Team, National Institute of Animal Science, Rural Development Administration, Wanju-gun, Republic of Korea; 2 Department of Animal Science and Technology, Konkuk University, Seoul, Republic of Korea; 3 Department of Agro-food Safety, National Institute of Agricultural Science, Rural Development Administration, Wanju-gun, Republic of Korea; 4 Monogastric Animal Feed Research Institute, Konkuk University, Seoul, Republic of Korea

**Keywords:** Pigs, Lead concentration, Prediction equation, Lead accumulation, Tissues

## Abstract

The objectives of this experiment were to determine the effects of graded dietary lead (Pb) concentrations on body weight and Pb concentrations in blood, hair, soft tissues, and urine from pigs and to generate equations for estimating daily Pb intake. Sixteen barrows with initial body weight 36.3 kg (standard deviation = 2.3) were allotted to four dietary treatments that consisted of graded supplemental Pb concentrations (0, 10, 25, and 250 mg/kg of diet). Daily feed allowances for each pig were 1 kg for first two weeks and 2 kg for last two weeks. The hair and blood of pigs were collected on d 14 and 28. At the end of experiment, the pigs were euthanized, and the liver, kidneys, muscle, and urine samples were collected. The prediction equations for estimating daily Pb intake of pigs were generated using Pb concentration of blood, hair, tissues, or urine as an independent variable. The Pb concentrations in the blood, hair, liver, kidneys, muscle, and urine linearly increased (*P* < 0.01) with increasing dietary Pb concentrations. There were quadratic effects (*P* < 0.05) of increasing dietary Pb concentration on Pb concentrations in the blood, hair, and muscle. There were highly positive correlations between dietary Pb concentration and Pb concentrations in the blood, hair, liver, kidneys, muscle, and urine (*r* > 0.83; *P* < 0.01). The equations were significant (*P* < 0.01) and showed high *r*^2^ (>0.83), except the equation using Pb concentration in the muscle as an independent variable. In conclusion, the dietary Pb concentration was highly correlated with Pb concentrations in the blood, hair, soft tissues, and urine of pigs. The total dietary Pb intake can be estimated from the Pb concentrations in the blood, hair, soft tissues, or urine for pigs.

## Introduction

Heavy metal poisoning in swine feeds is one of the problems in the swine industry. Swine feeds can be contaminated through mineral premix and ingredients originated from soil containing high concentration of heavy metals (Obeid et al., 2014; MacLachlan et al., 2015). The heavy metal fed to pigs can depress growth performance and cause clinical disorders. The heavy metals can be accumulated in soft tissues, bones, and hair of pigs and accumulated heavy metals in edible tissues of pigs can be transferred to human and other animals through the food chain (Doyle & Spaulding, 1978; Grybauskait, Laurinavicien & Cikockait, 2014; Obeid et al., 2014).

Domestic animals are potentially exposed to lead (Pb) via contaminated mineral premix, and contaminated feed ingredients and soil (MacLachlan et al., 2015). Adverse metabolic effects occur in animals when they are fed Pb, and the Pb is extremely toxic with relatively small amounts (Doyle, 1979). The Pb can be absorbed through the gastrointestinal tract, respiratory tract, and skin, and then can be accumulated in various animal tissues. However, absorbed Pb is excreted very slowly from the body (Papanikolaou et al., 2005).

The Pb concentrations in hair and teeth of pigs have been reported to be good indicators of Pb accumulation (Phillips, Győri & Kovács, 2003). To the best of our knowledge, however, there is very limited information on the relationship between the dietary Pb intake and accumulated Pb concentration in tissues of pigs. Therefore, the objectives of this experiment were to investigate the influence of graded supplemental Pb concentrations on body weight (BW) and Pb concentrations in blood, hair, soft tissues (liver, kidneys, and muscle), and urine from pigs and to generate equations for estimating daily Pb intake based on Pb concentration in blood, hair, soft tissues, or urine as an independent variable for pigs.

## Materials and Methods

The protocol for the animal experimental procedures was reviewed and approved by the Institutional Animal Care and Use Committee of the National Institute of Animal Science (No. 2015-147).

### Animals, diets, and experimental design

Sixteen barrows with a mean initial BW of 36.3 kg (standard deviation = 2.3) were randomly allocated to four dietary treatments with four replications per each treatment. The piglets were individually housed in the pen (2.1 m × 1.4 m). Initially, the piglets were allowed to adjust for one week (wk) to their new housing at 25 ± 1 °C. Then the pigs were fed the assigned experimental diet for four weeks with 1 kg per day for first two weeks and 2 kg per day for last two weeks. The four dietary treatments with four concentrations of supplemental Pb (0, 10, 25, and 250 mg/kg of diet) were prepared. The control diet without supplemental Pb was formulated based on corn and soybean meal to meet the nutrient requirements for swine given by US National Research Council (National Research Council, 2012; Table 1). For convenience of feeding the Pb to the pigs, the supplemental Pb as a lead sulfate was weighed and encapsulated into two separated capsules. The amount of supplemental Pb was calculated based on the daily feed allowance and target dietary Pb concentration (10, 25, or 250 mg/kg of diet). The encapsulated Pb was supplemented to the treatment groups along with the diet at each meal. All pigs were fed the diets twice daily and were allowed free access to water. The health status of piglets was observed during overall experimental period.

**Table 1:** Ingredients and chemical compositions of control diet (as-fed basis).

Item	Control diet
Ingredients (%)	
Ground corn	58.56
Soybean meal (46% crude protein)	14.00
Extruded soybean meal	12.00
Whey powder (12% crude protein)	7.00
Fish meal	3.45
Soybean oil	1.60
_L_-Lysine ⋅HCl (78%)	0.43
_DL_-Methionine (99%)	0.14
_L_-Threonine (99%)	0.12
Monodicalcium phosphate	1.08
Ground limestone	0.60
Choline chloride (50%)	0.20
Sodium chloride	0.32
Vitamin-trace mineral premix^a^	0.50
Calculated nutrients (%)	
Metabolizable energy (kcal/kg)	3,444
Crude protein	20.78
Lysine	1.47
Methionine	0.49
Crude fiber	2.29
Calcium	0.75
Phosphorus	0.45

**Notes.**

a Provided the following quantities per kg of complete diet: vitamin, A 11,000 IU; vitamin D_3_, 1,500 IU; vitamin E, 44.1 IU; vitamin K_3_, 4.0 mg; vitamin B_1_, 1.4 mg; vitamin B_2_, 5.22 mg; vitamin B_5_, 20.0 mg; vitamin B_12_, 0.01 mg; niacin, 26.0 mg; pantothenic acid, 14 mg; folic acid, 0.8 mg; biotin, 44 µg; Fe, 100.0 mg as iron sulfate; Cu, 16.50 mg as copper sulfate; Zn, 90.0 mg as zinc sulfate; Mn, 35.0 mg as manganese sulfate; I, 0.30 mg as calcium iodate.

### Sampling and processing

The BW of pigs was measured on d 0, 7, 14, 21, and 28 in the morning before feeding. The hair and blood samples of each pig were collected on d 14 and 28. Blood samples were taken by jugular venipuncture into metal free heparinized collection tubes (BD Vacutainer^®^ 366480) to avoid error of analysis, and immediately stored at −20 °C for until analysis. At the end of the experimental period, all control and dietary treatment groups were euthanized by an anesthetic overdose with the pentobarbital. It is a barbiturate and act as a central nervous system depressant. After the pigs were slaughtered, urine samples were collected directly from the urinary bladder by using syringe. The liver, both side of kidneys, and muscle tissues were collected immediately and rapidly frozen in liquid nitrogen, and stored at −80 °C for further analysis.

**Table 2:** Final body weight (BW) and weight of liver and kidneys for pigs fed the diets containing graded concentrations of supplemental lead (Pb).^a^

	Supplemental Pb, mg/kg of diet					*P*-value	
Item	0	10	25	250	SEM^b^	Linear	Quadratic
Final BW, kg	57.5	57.9	56.5	58.8	1.5	0.423	0.559
Weight, g							
Liver	1,983	1,823	1,928	1,723	90	0.091	0.867
Kidneys	315	315	333	365	30	0.208	0.750
Relative weight to BW, %							
Liver	3.45	3.16	3.42	2.94	0.18	0.070	0.880
Kidneys	0.55	0.55	0.59	0.62	0.05	0.355	0.611

**Notes.**

a Each least squares mean represents four observations.

b SEM, standard error of the means.

**Table 3:** Lead (Pb) concentrations in blood, hair, liver, kidneys, muscle, and urine for pigs fed the diets containing graded concentrations of supplemental Pb.^a^

	Supplemental Pb (mg/kg of diet)					*P*-value	
Item	0	10	25	250	SEM^b^	Linear	Quadratic
Blood (mg/L)							
d 14	0.03	0.06	0.09	0.26	0.01	<0.001	0.026
d 28	0.01	0.07	0.12	0.52	0.02	<0.001	0.020
Hair (mg/kg)							
d 14	0.02	0.87	1.19	2.80	0.03	<0.001	<0.001
d 28	0.09	1.28	2.12	9.89	0.08	<0.001	<0.001
Liver (mg/kg)	0.42	4.03	9.74	90.29	2.00	<0.001	0.903
Kidneys (mg/kg)	0.53	6.26	13.44	117.89	3.97	<0.001	0.834
Muscle (mg/kg)	0.58	1.85	2.59	3.99	0.17	<0.001	<0.001
Urine (mg/L)	0.01	0.02	0.05	0.43	0.01	<0.001	0.917

**Notes.**

a Each least squares mean represents four observations.

b SEM, standard error of the means.

### Sample digestion procedure and lead concentration analysis

The liver, kidneys, and muscle tissues were dried at 105 °C for 24 h to stable weight (Linden, Olsson & Oskarsson, 1999) and ground to a fine powder by using mortar and pestle. The samples were immediately taken into a small sample bottle and tightened the cap for avoiding moisture absorption. The amounts of samples for analyzing Pb concentration were 1 mL of blood sample, 0.1 g for hair sample, 0.2 g of organ and muscle samples, and 1.5 mL of urine samples based on the expected Pb concentrations. Before digestion, the hair samples were washed according to the method reported by Chaturvedi et al. (2014). The Pb concentration analysis for each sample was performed in triplicate, and three blank samples were also analyzed for each digestion.

**Figure 1:**
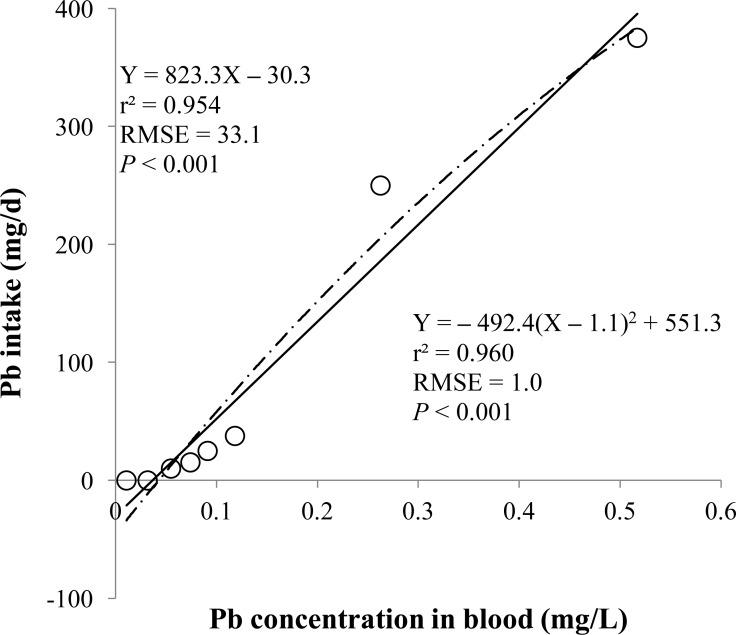
Blood. Linear and quadratic regression equations for estimating lead (Pb) intake (mg/d) based on Pb concentration in blood (mg/L) on d 14 and 28. Each data point represents least squares mean of four observations. *r*^2^, coefficient of determination; RMSE, root mean square of error.

**Table 4:** Correlation coefficients (*r*) between supplemental lead (Pb) concentration and final body weight (BW), liver and kidneys weights relative to BW, and Pb concentration in blood, hair, liver, kidneys, muscle, and urine.

	*r*	
Item	Supplemental Pb concentration	*P*-value
Final BW (kg)	0.228	0.396
Liver weight relative to BW	−0.473	0.064
Kidneys weight relative to BW	0.264	0.323
Pb concentration		
Blood (mg/L)		
d 14	0.969	<0.001
d 28	0.985	<0.001
Hair (mg/kg)		
d 14	0.935	<0.001
d 28	0.994	<0.001
Liver (mg/kg)	0.996	<0.001
Kidneys (mg/kg)	0.990	<0.001
Muscle (mg/kg)	0.835	<0.001
Urine (mg/L)	0.998	<0.001

The samples were digested and analyzed based on the procedure adapted from Ashoka et al. (2009). The samples were placed in a screw cap Pyrex glass tube, and 2.5 mL of concentrated HNO_3_ and 0.5 mL of concentrated HCl were added. The cap was tightened and the tubes were kept in a water bath at 85 °C for 3 h. After digestion, all the tubes were kept at room temperature for cooling, filtered (syringe filter, 0.20 µm pore diameter, Hyundai Micro Co., LTD, Seoul, Republic of Korea), and then diluted to 50 mL with a 2% HNO_3_ solution in a volumetric flask to compensate for any ‘acid effect’ (Cubadda, Raggi & Coni, 2006). The Pb concentration in the samples was analyzed using an inductively coupled plasma source mass spectrometer (Agilent 7500; Agilent, Santa Clara, CA, USA).

**Figure 2:**
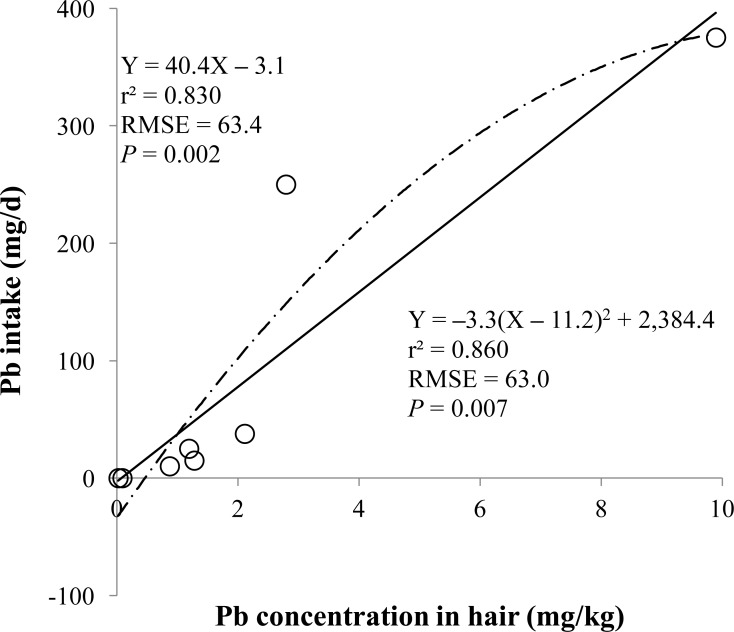
Hair. Linear and quadratic regression equations for estimating lead (Pb) intake (mg/d) based on Pb concentration in hair (mg/kg) on d 14 and 28. Each data point represents least squares mean of four observations. *r*^2^, coefficient of determination; RMSE, root mean square of error.

**Figure 3:**
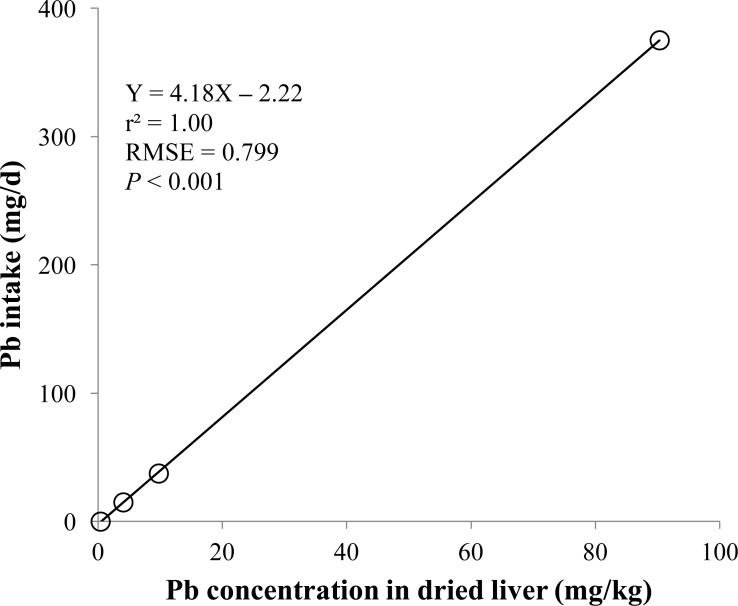
Dried liver. Linear regression equation for estimating total lead (Pb) intake (mg/d) based on Pb concentration in dried liver (mg/kg). Each data point represents least squares mean of four observations. *r*^2^, coefficient of determination; RMSE, root mean square of error.

### Statistical analysis

Experimental data were analyzed using the MIXED procedure of SAS (SAS Institute Inc., Cary, NC, USA). The model included the dietary Pb concentration as the independent variable. The orthogonal polynomial contrasts were used to examine linear and quadratic effects of dietary Pb concentrations. The IML procedure of SAS was used to generate the contrast coefficients for unequally spaced dietary Pb concentrations. The correlation between dietary Pb concentration and response criteria was determined using CORR procedure of SAS. To estimate daily Pb intake of pigs based on the Pb concentrations in organs and tissues, linear and quadratic equations were developed using the REG and NLIN procedures of SAS, respectively. The experimental unit was the pig and statistical significance was determined at *P* < 0.05.

## Results

There were no linear and quadratic effects on the final BW, weight of liver and kidneys, and relative weight to BW of liver and kidneys with increasing dietary Pb concentration (Table 2). The Pb concentrations in the hair, blood, and muscle linearly and quadratically increased (*P* < 0.05) as the supplemental Pb concentration increased (Table 3) both on d 14 and 28. The Pb concentrations in the liver, kidneys, and urine were linearly increased (*P* < 0.01) with increasing dietary Pb concentration.

The correlation coefficients (*r*) between the dietary Pb concentration and the response criteria were determined (Table 4). There was no significant correlation between the dietary Pb and final BW. The Pb concentrations in the blood, hair liver, kidneys, muscle, and urine were strongly and positively correlated with the dietary Pb concentration (*r* > 0.84, *P* < 0.01).

**Figure 4:**
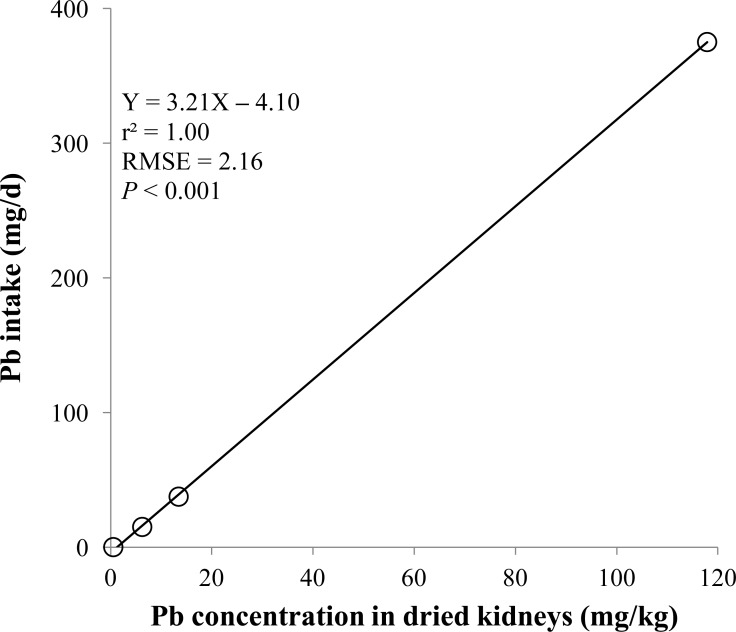
Dried kidneys. Linear regression equation for estimating lead (Pb) intake (mg/d) based on Pb concentration in dried kidneys (mg/kg). Each data point represents least squares mean of four observations. *r*^2^, coefficient of determination; RMSE, root mean square of error.

**Figure 5:**
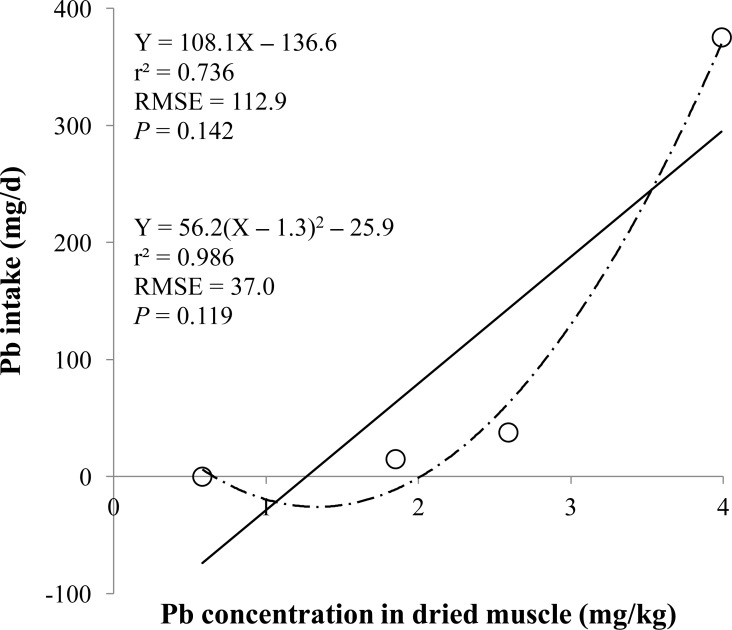
Dried muscle. Linear and quadratic regression equations for estimating lead (Pb) intake (mg/d) based on Pb concentration in dried muscle (mg/kg). Each data point represents least squares mean of four observations. *r*^2^, coefficient of determination; RMSE, root mean square of error.

**Figure 6:**
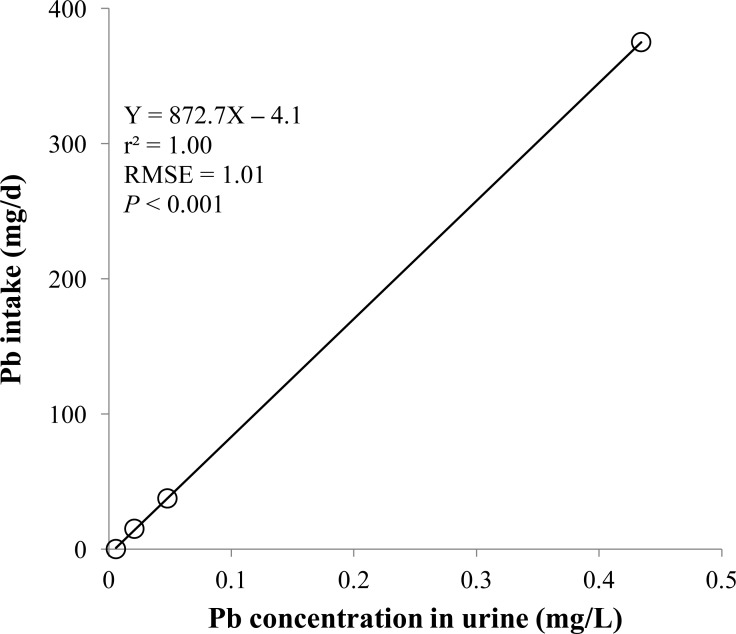
Urine. Linear regression equation for estimating lead (Pb) intake (mg/d) based on Pb concentration in urine (mg/L). Each data point represents least squares mean of four observations. *r*^2^, coefficient of determination; RMSE, root mean square of error.

The prediction equations for the daily Pb intake were generated based on the Pb concentration in the blood, hair, liver, kidneys, muscle, or urine. The linear and quadratic equations generated using the Pb concentrations in the blood and hair were significant (*P* < 0.01; Figs. 1 and 2) with high *r*^2^ (>0.83). The linear equations generated using the Pb concentrations in the liver and kidneys were significant (*P* < 0.01; Figs. 3 and 4) with high *r*^2^ (1.00). The linear and quadratic equations generated using the Pb concentration in the muscle were not significant (Fig. 5). The linear equations generated using the Pb concentrations in the urine were also significant (*P* < 0.01; Fig. 6) with high *r*^2^.

## Discussion

Lead is one of toxic heavy metals to human and animals, and can be toxic to animals in very small quantities (Doyle, 1979). The Pb concentration in livestock diets is regulated (less than 10 mg/kg in Republic of Korea and 5 mg/kg in EU and Canada). While animal feed ingredients and diets generally contain much less than 1 mg/kg of Pb, mineral sources and premix may have relatively high Pb concentration (Nicholson et al., 1999; MacLachlan et al., 2015; Dai et al., 2016). Feed processing procedures and environmental pollution can increase the Pb concentration in animal feeds (Wang et al., 2013). Therefore, careful and continuous monitoring of Pb concentrations in feed ingredients is needed.

In the current experiment, there were no linear and quadratic effects on the final BW with increasing the dietary Pb concentration. In previous studies, the inclusion of Pb in the swine diets decreased the growth performance of pigs (Hsu et al., 1975; Phillips, Győri & Kovács, 2003). The lack of response in the BW in the present work may be due to the relatively short feeding period compared with the previous studies. In addition, the pigs were *ad libitum* fed in the previous studies. However, in the current experiment, the daily feed intake of each pig was restricted in 1 kg or 2 kg in overall experimental period. Zacharias, Lantzsch & Drochner (1999) also reported that there were no effects of dietary Pb on the growth performance in restricted feeding condition. The restricted feeding may cause the inconsistent results for growth performance among the studies. The different initial BW of pigs among the studies might affect the results of the experiments. The initial BW of pigs in the previous studies was from 7.5 to 17 kg (Hsu et al., 1975; Zacharias, Lantzsch & Drochner, 1999; Phillips, Győri & Kovács, 2003). Younger animals can absorb more dietary Pb from the gastrointestinal tract than adult animals do (Doyle & Spaulding, 1978). The toxicity of Pb in pigs varies depending on nutritional status and age as well as on the chemical form, dosage, and experimental duration (Doyle & Spaulding, 1978; Papanikolaou et al., 2005).

The absorption of Pb depends on the form of Pb, route of administration, age, and physiological state. The accumulated concentrations of Pb are the highest usually in bone, kidneys, liver, aorta, and hair, while the lowest in muscle, adipose tissues, and brain (Quarterman, 1986). There are three compartments of absorbed Pb: blood, soft tissues, and skeleton (Quarterman, 1986). In the current experiment, the Pb concentrations in all samples of blood, hair, and soft tissues increased with increasing dietary Pb concentration. Absorbed Pb is mainly accumulated in the liver and kidneys (Doyle & Spaulding, 1978), and the liver and kidneys showed a fast Pb accumulation rate in pigs (Zacharias, Lantzsch & Drochner, 1999; Phillips, Győri & Kovács, 2003). Although the majority of ingested Pb is excreted in feces without absorption, the main excretion route of absorbed Pb is urinary tract (Papanikolaou et al., 2005). This may be a reason for the increment in the Pb concentrations in the urine and kidneys with increasing dietary Pb concentration in this study.

In the previous study, the dietary Pb concentration was reflected in the Pb concentration found in the kidneys, hair, and teeth (Phillips, Győri & Kovács, 2003). In the current study, the equations for predicting the daily Pb intake using the Pb concentrations in the different tissues were generated, and the equations had the *r*^2^ greater than 0.83. The Pb concentrations in the blood, hair, liver, and kidneys samples were good indicators for estimating daily Pb intake. The results of this study confirmed that the blood and hair can be used as an antemortem biomarker for Pb contamination.

In conclusion, the dietary Pb intake was highly correlated with Pb concentrations in the blood, hair, soft tissues (liver, kidneys, and muscle), and urine samples of pigs. The daily Pb intake can be estimated from the Pb concentrations of various tissues of pigs including blood, hair, soft tissues, or urine from pigs. Further research will be needed to investigate the factors affecting the Pb accumulations in pigs including the BW and duration.

##  Supplemental Information

10.7717/peerj.3936/supp-1 Data S1 Raw data

## Additional Information and Declarations

### Data Availability

The following information was supplied regarding data availability: The raw data has been supplied as Data S1.
